# Corrrelation of the Specific Rates of Solvolysis of Ethyl Fluoroformate Using the Extended Grunwald-Winstein Equation

**DOI:** 10.3390/ijms10030929

**Published:** 2009-03-02

**Authors:** Mi Hye Seong, Jin Burm Kyong, Young Hoon Lee, Dennis N. Kevill

**Affiliations:** 1 Department of Chemistry and Applied Chemistry, Hanyang University, Ansan-si, Gyeonggi-do 426-791, Korea; 2 Department of Food and Biotechnology, Hanseo University, Seosan, ChungNam, 356-706, Korea; E-Mail: chemtec@hanseo.ac.kr; 3 Department of Chemistry and Biochemistry, Northern Illinois University, DeKalb, Illinois 60115-2862, USA

**Keywords:** Ethyl fluoroformate, addition–elimination, Grunwald–Winstein equation, solvent isotope effect

## Abstract

The specific rates of solvolysis of ethyl fluoroformate have been measured at 24.2 °C in 21 pure and binary solvents. These give a satisfactory correlation over the full range of solvents when the extended Grunwald-Winstein equation is applied. The sensitivities to changes in the *N*_T_ solvent nucleophilicity scale and the *Y*_Cl_ solvent ionizing power scale, and the k_F_/k_Cl_ values are very similar to those for solvolyses of *n*-octyl fluoroformate, consistent with the addition step of an addition-elimination pathway being rate-determining. For methanolysis, a solvent deuterium isotope effect of 3.10 is compatible with the incorporation of general-base catalysis into the substitution process. For five representative solvents, studies were made at several temperatures and activation parameters determined. The results are also compared with those reported earlier for ethyl chloroformate and mechanistic conclusions are drawn.

## 1. Introduction

The extended Grunwald-Winstein Equation (1) can give information which is very helpful in assessing the mechanism of solvolysis reactions [1–4]. In Equation (1), k and k_o_ are the specific rates of solvolysis of a substrate in a given solvent and in 80% ethanol, respectively; *l* is the sensitivity towards changes in *N*_T_, a scale of solvent nucleophilicity based on the specific rates of solvolysis of the *S*-methyldibenzothiophenium ion; and *m* is the sensitivity towards changes in *Y*_Cl_, a scale of solvent ionizing power based on the specific rates of solvolysis of 1-adamantyl chloride.

(1) $$\text{log}\;(\text{k}/{\text{k}}_{\text{o}})=lN_{\text{T}}+mY_{\text{Cl}}+c$$

Although both the *N*_T_ and *Y*_Cl_ scales are based on standard systems involving substitution reaction at an sp^3^-hybridized carbon [3,4], the scales have also been used with considerable success in the correlation analyses of solvolysis reactions of substrates which have attack at the sp^2^-hybridized carbon of acyl halides [5–7] and chloroformate esters [8–15], and at the sulfur atom of sulfonyl halides [16].

Alkyl halogenoformate esters are important reagents which are widely used in physiological and biological studies [17,18]. A recently published study of the solvolysis of ethyl chloroformate (EtOCOCl) [19] is extended to ethyl fluoroformate (EtOCOF). Although the solvolyses of alkyl and aryl haloformates have been extensively studied kinetically, less is known about the kinetics and mechanism of the solvolyses of fluoroformates. Accordingly, a study of the mechanism of the reactions of fluoroformates under solvolytic conditions is of continuing interest.

We reported that the specific rates of solvolysis of ethyl chloroformate (EtOCOCl) [19] can be very well correlated, after division into two solvent groups, using the extended Grunwald-Winstein equation [Equation (1)], with an *l*-value of 1.56±0.09 and *m*-value of 0.55±0.03 (correlation coefficient of 0.967) in the less ionizing and more nucleophilic solvents, and with an *l*-value of 0.69±0.13 and *m*-value of 0.82±0.16 (correlation coefficient of 0.946) in the more ionizing and least nucleophilic solvents (HCO_2_H, 100% and 97% TFE and 97% ~50%HFIP).

(2) $$\begin{array}{c} \text{EtOCOCl}\;+\;2\text{SOH}\;\to \;\text{EtOCOOS}\;+\;\text{SO}{\text{H}}_{2}^{+}\;+\;\text{C}{\text{l}}^{-}\\ \text{EtOCOOH}\;\to \;\text{EtOH}\;+\;\text{C}{\text{O}}_{2}\;(\text{when}\;\text{S}=\text{H})\\ \left[\text{Addition-Elimination}\;\text{Mechanism}\right] \end{array}$$

**Figure:**
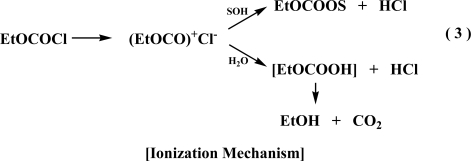

The solvolyses of ethyl chloroformate can be expressed according to Equations (2) and (3), with an addition-elimination mechanism [Equation (2)] involving substitution at the acyl carbon in the less ionizing and more nucleophilic solvents and an ionization mechanism [Equation (3)] in the more ionizing and least nucleophilic solvents.

Previous work concerning the solvolyses of *n*-octyl haloformates [20] found the k_F_/k_Cl_ ratio to be only slightly less than unity in 100% ethanol and 100% methanol and to be somewhat above unity for solvolyses in mixtures of water with ethanol, acetone, dioxane, or 2,2,2-trifluoroethanol (TFE). These ratios were considered to be consistent with the addition step of an addition-elimination mechanism being rate determining. For solvolyses of ethyl haloformates, k_F_/k_Cl_ ratios of 5.46 for 70% acetone at 30.1 °C [21] and of 1.1 for 100% ethanol at 25.1 °C [22] have been reported.

In the present study, we report on the specific rates for solvolyses of ethyl fluoroformate in a wide range of solvent types. Mechanistic conclusions are then drawn from a consideration of the analyses using the extended Grunwald-Winstein equation, including a comparison with the *l* and *m*-values previously determined from kinetic studies of other haloformate esters. In addition to a detailed extended Grunwald-Winstein equation treatment of the specific rates, the influence of temperature on the specific rate (for three and four solvents) allows enthalpies and entropies of activation to be calculated and a measurement in methanol-*d* allows a determination of the solvent deuterium isotope effect. These analyses are also combined with a consideration of leaving-group effects to arrive at a reasonable mechanism.

## 2. Results

The specific rates of solvolysis of ethyl fluoroformate at 24.2 °C are reported in Table 1. The solvents consisted of ethanol, methanol, binary mixtures of water with ethanol, methanol, 2,2,2-trifluoroethanol (TFE), acetone, and 1,1,1,3,3,3-hexafluoro-2-propanol (HFIP) and four binary mixtures of TFE and ethanol. The required *N*_T_ and *Y*_Cl_ values are also reported in Table 1, together with the k_F_/k_Cl_ ratios. A determination was also made in methanol-*d* (MeOD). In methanol, ethanol, 80% ethanol, 70% TFE, and 70% HFIP, specific rates of solvolysis of ethyl fluoroformate and chloroformate were determined at two and three additional temperatures, and these values, together with calculated enthalpies and entropies of activation, are reported in Table 2.

## 3. Discussion

The variance of k_F_/k_Cl_ ratios has suggested [23] differences in mechanism and a useful additional probe will be to apply the extended Grunwald-Winstein equation (Equation 1) and compare the *l* and *m* values with those previously obtained for alkyl fluoroformates. Although some authors [24,25] claim that leaving group effects in solvolytic reactions are not very sensitive to mechanistic changes, the consideration of these effects in nucleophilic substitution reactions has long been recognized as a useful tool in studying the reaction mechanism [26].

For S_N_1 reaction, a value as low as 10^−7^ was observed in 4-(*N,N*-dimethylamino)benzoyl halide solvolyses [27] and a low value of 1.3×10^−4^ was also observed for acetyl halide solvolyses in 75% acetone [26]. These values reflect an appreciable ground-state stabilization for the fluoride [28] and the need to break a strong carbon–fluorine bond in the rate determining step. In contrast, if the addition step is rate-determining, values of close to unity (and frequently above it), reflecting a large electron deficiency at the carbonyl carbon of a haloformate incorporating fluorine [7], are frequently observed. This situation has recently been discussed in a consideration of *n*-octyl haloformate solvolyses [20], where k_F_/k_Cl_ specific rate ratios of 0.6 to 15 were observed. Similar ratios of k_F_/k_Cl_ specific rates have been observed previously for the solvolyses of other haloformate esters [14,20,29,30]. For other haloformate esters, k_F_/k_Cl_ ratios of 1.09 to 7.16 for solvolyses in 70% aqueous acetone at 30.1°C have been reported [21].

The leaving group specific rate ratios (k_F_/k_Cl_) determined in the present study for ethyl haloformate are compared with the specific rate ratios for the same leaving groups observed in the bimolecular pathway of *n*-propyl, *i*-propyl, *n*-octyl, benzyl, and 1- and 2-adamantyl haloformates in various solvents [20,31–33] (Table 3). The specific rate ratios (k_F_/k_Cl_) for the solvolyses of ethyl fluoroformate and chloroformate are similar to the values for all the other primary and secondary substrates but significantly larger than the analogous specific rate ratios for the partially solvolysis-decomposition reaction (ionization pathway) of 1-adamantyl fluoroformate relative to the chloroformate in methanol, ethanol, and 80% ethanol. In these solvents, essentially all the reaction of 1-adamantyl chloroformate is by the ionization pathway.

The solvent deuterium isotope effect value (footnote to Table 1) for methanolysis of ethyl fluoroformate of k_MeOH_/k_MeOD_ = 3.10±0.24 at 24.2 °C is of a magnitude usually taken to indicate that nucleophilic attack by a methanol molecule is assisted by general-base catalysis by a second methanol molecule [13, 34, 35]. The solvent deuterium isotope effect value of ethyl fluoroformate is higher than for the methanolysis of ethyl chloroformate (k_MeOH_/k_MeOD_ = 2.22±0.24 at 45.0 °C) or for the ethanolysis of a series of *para*-substituted phenyl chloroformates, where values in the range of 2.1~2.5 were obtained [36, 37]. The higher value gives further support for the proposal that bond formation is more advanced at the transition state for addition to fluoroformates than for chloroformates.

For five solvents, the values of the enthalpy and the entropy of activation for the solvolysis of ethyl fluoroformate are 9.4 ~ 14.0 Kcal/mol and −27.9 ~ −41.5 cal/mol·K, and the values for the solvolysis of ethyl chloroformate are 13.6 ~ 18.4 Kcal/mol and −20.4 ~ −31.8 cal/mol·K, respectively (Table 2). The very negative entropies of activation are consistent with the bimolecular nature of the proposed rate-determining step. While a bimolecular mechanism for the solvolyses is strongly indicated by the much slower reactions in TFE-rich solvents and by the appreciably negative entropies of activation, it is not established whether the process involves a stepwise addition-elimination (association-dissociation) or a concerted (S_N_2) pathway. A powerful test in considering detailed mechanisms of solvolysis is to carry out a correlation analysis using the extended Grunwald-Winstein equation (Equation 1).

The specific rates of solvolysis of ethyl fluoroformate were studies in a wide variety of pure and binary solvents, including the TFE- and HFIP-containing systems, which are important components of the extended Grunwald-Winstein correlations.

**Figure 1.**
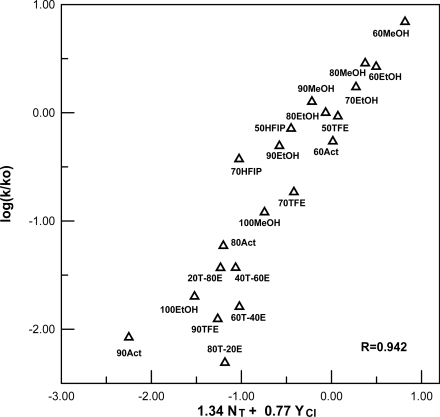
Plot of log (k/k_o_) for solvolyses of ethyl fluoroformate at 24.2°C against (1.34*N*_T_ + 0.77*Y*_Cl_). The data points for TFE-ethanol mixtures are not included in the correlation.

To see the effect of including fluoroalcohol-containing solvents in the correlation of the specific rates of solvolysis of ethyl fluoroformate, we have included 9 solvents with a fluoroalcohol (TFE or HFIP) component. As can be seen in Figure 1, there are appreciable deviations from the plot for the solvolytic data in TFE-ethanol mixtures with the largest deviations for the 80% TFE-20% ethanol and 60%TFE-40% ethanol points. Also in earlier correlations of other haloformate esters, it was found that the data points for these solvent systems usually lay below the correlation line [10,14,30,38].

Correlations were carried out both with and without the TFE-ethanol data. An analysis of the data using the extended Grunwald-Winstein equation to the specific rates of solvolysis of ethyl fluoro-formate leads to a poor linear correlation with values of 1.51 ± 0.20 for *l*, 0.85 ± 0.11 for *m,* −0.14 ± 0.14 for *c*, and 0.883 for the correlation coefficient. Recalculation with omission of the four data points in TFE-ethanol mixtures led to values of 1.34±0.14 for *l*, 0.77±0.07 for *m*, −0.06±0.10 for c, and 0.942 for the correlation coefficient. When the TFE-ethanol points are omitted from the correlation, the *l* and *m* values are only slightly reduced but a considerably improved value for correlation coefficient (0.942 relative to 0.883) is observed. The results of the correlation are reported in Table 4, together with the corresponding parameters obtained in the analyses of earlier studied substrates. The higher *m*-values for the solvolyses of fluoroformates, relative to chloroformates, may reflect the kinetically favorable influence of increased solvation of the developing negative charge on the carbonyl oxygen in the presence of the more electronegative fluorine attached at the carbonyl carbon.

The *l/m* ratio has been suggested as a useful mechanistic criterion and the values of Table 4 divide nicely into two classes with values of 1.8 to 2.8 for those entries postulated to represent addition-elimination (A-E) and 0.54 to 0.84 for those believed to represent ionization (I).

The *l* and *m* values of ethyl fluoroformate in Table 4 are very similar to those for the earlier studied substrates (*n*-propyl-, *i*-propyl-, and *n*-octyl fluoroformates), which have been shown to solvolyze with the addition step of an addition-elimination pathway being rate determining.

**Figure 2.**
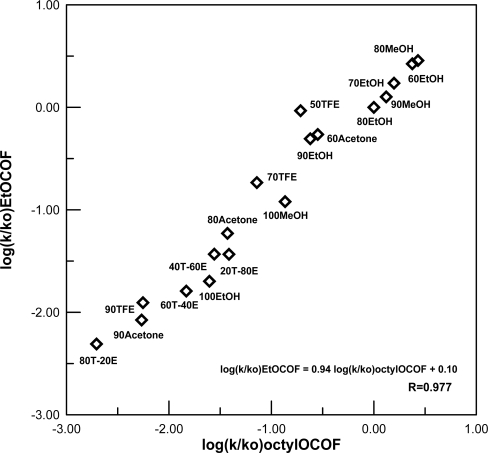
Plot of log (*k/k_o_*) for solvolyses of ethyl fluoroformate against log (*k/k_o_*) for solvolyses of *n*-octyl fluoroformate at 24.2 °C.

To prove further the similarity between solvent effects upon the specific rates of solvolysis of ethyl-and *n*-octyl fluoroformates, we have carried out a direct comparison of the log (k/k_o_) values for ethyl fluoroformate against those for *n*-octyl fluoroformate for the 18 solvents for which data in available for both substrates. A good linear plot (Figure 2) was obtained, with a slope of 0.94 ± 0.06, intercept of 0.10± 0.09, and correlation coefficient of 0.977.

## 4. Conclusions

The solvolyses of ethyl fluoroformate give a satisfactory extended Grunwald-Winstein correlation [Equation (1)] over wide range of *N*_T_ and *Y*_Cl_ values. The sensitivities to changes in *N*_T_ and *Y*_Cl_ (*l*=1.34 and *m*=0.77) are very similar to those for the several fluoroformate esters (Table 4), which are shown to solvolyze with the addition step of an association-dissociation (addition-elimination) pathway being rate determining.

The k_F_/k_Cl_ values obtained in a comparison with the corresponding solvolysis of ethyl chloroformate are very similar to those for solvolyses of *n*-octyl fluoroformate, consistent with a bimolecular addition-elimination mechanism, proceeding through a tetrahedral intermediate.

The solvent deuterium isotope effect value for methanolysis (k_MeOH_/k_MeOD_) of 3.10 is of a magnitude usually taken to indicate that nucleophilic attack by a methanol molecule is assisted by general-base catalysis by a second methanol molecule.

The entropies of activation (−27.9 ~ −41.5 cal/mol·K) for ethyl fluoroformate solvolyses believed to involve rate-determining attack at acyl carbon are considerably more negative than the values for solvolyses believed to proceed by the ionization pathway (the entropies of activation are for 1-adamantyl chloroformate +3.3 ~ +6.7 cal/mol·K [29] and for 1-adamantyl fluoroformate −8.0 ~ −14.7 cal/mol·K [30]). The more negative entropy of activation values for the ethyl fluoroformate reaction are consistent with the bimolecular nature of the rate-determining step.

In the present study, unlike the solvolyses of ethyl chloroformate [Equations (2) and (3)], where the two reaction channels (addition-elimination and ionization pathways) were observed, the solvolyses of ethyl fluoroformate have a pathway involving bimolecular attack by solvent at acyl carbon, with what is suggested to be the addition step of an addition-elimination pathway being rate determining [Equation (2)].

## 5. Experimental

The ethyl chloroformate (Aldrich) was purified by fractional distillation under reduced pressure. The ethyl chloroformate (22.7g, 0.209 mol) was syringed into a three-neck flask (200 mL) containing dried KF (15.9g, 0.274 mol) and 18-crown-6 (1.95g, 0.00736 mol) and fitted with a Teflon stirring bar, a condenser topped by an Ar gas inlet, a septum cap, and a ground glass stopper, as described earlier [40]. The mixture then was stirred efficiently at room temperature until FT-IR (Bio-Rad FTS 6000) analysis of an aliquot indicated that no chloroformate remained (C=O stretch at 1777 cm^−1^; fluoroformate C=O stretch at 1837 cm^−1^). After a reaction time of 68 hours, the product fluoroformate was isolated directly from the reaction apparatus by simple distillation at a reaction temperature of 56–57 °C (lit. [41] 55–57 °C).

Solvents were purified as previously described [31]. The kinetic procedures were as described earlier [30, 31], using a substrate concentration of about 5.86 × 10^−3^ *M* and with 5 mL aliquots removed for titration. The *l* and *m* values were calculated using multiple regression analysis.

## Figures and Tables

**Table 1.** Specific rates of solvolysis (with standard deviation) of ethyl fluoroformate *a* in pure and binary solvents at 24.2 °C together with the appropriate solvent nucleophilicity (***N*_T_**) and solvent ionizing power (***Y*_Cl_**) values and the specific rate ratio relative to ethyl chloroformate (**k_F_ / k_Cl_**).

Solvent(%)*b*	10^4^ k, s^−1^	*N*_T_*c*	*Y*_Cl_*d*	k_F_ /k_Cl_^e^
100 MeOH *f*	0.769±0.040	0.17	−1.17	0.93
90 MeOH	8.09±0.47	−0.01	−0.18	4.82
80 MeOH	18.3±0.8	−0.06	0.67	7.43
60 MeOH	44.2±3.1	−0.54	2.07	11.1
100 EtOH	0.128±0.010	0.37	−2.52	0.57
90 EtOH	3.15±0.09	0.16	−0.94	5.77
80 EtOH	6.39±0.06	0.00	0.00	8.74
70 EtOH	11.0±0.7	−0.20	0.78	11.1
60 EtOH	17.0±0.4	−0.38	1.38	14.0
90Me_2_CO	0.0536±0.0023	−0.35	−2.22	1.80
80 Me_2_CO	0.376±0.022	−0.37	−0.83	3.90
60 Me_2_CO	3.47±0.22	−0.52	0.95	9.44
90TFE*g*	0.0794±0.0016	−2.55	2.85	13.3
70 TFE*g*	1.18±0.04	−1.98	2.96	19.3
50 TFE*g*	5.92±0.56	−1.73	3.16	28.1
80T-20E*h*	0.0313±0.0010	−1.76	1.89	5.03
60T-40E*h*	0.103±0.008	−0.94	0.63	3.47
40T-60E*h*	0.236±0.023	−0.34	−0.48	2.85
20T-80E*h*	0.235±0.019	0.08	−1.42	1.65
70HFIP*g*	2.38±0.19	−2.94	3.83	53.6
50HFIP*g*	4.55±0.35	−2.49	3.80	33.2

^a^ Substrate concentration of 5.86×10^−3^ mol dm^−3^ .

^b^ Unless otherwise indicated, on a volume/volume basis, at 25.0 °C, with the other component water.

^c^ Values from ref. [3].

^d^ Values from ref. [4].

^e^ Values relative to those for the corresponding solvolysis of ethyl chloroformate (values from ref. [19]).

^f^ Value in 100% MeOD of 0.248(±0.014)×10^−4^ s^−1^, leading to a k_MeOH_/k_MeOD_ value of 3.10±0.24, and specific rates of solvolysis of ethyl chloroformate in 100% MeOH and MeOD at 45.0 °C is (4.59±0.16)_MeOH_×10^−4^ s^−1^ and (2.07±0.01)_MeOD_×10^−4^ s^−1^, respectively and k_MeOH_/k_MeOD_ value of solvolysis of ethyl chloroformate is 2.22±0.09.

^g^ Solvent prepared on weight/weight basis.

^h^ T–E represents 2,2,2-trifluoroethanol–ethanol mixtures.

**Table 2.** Specific rates for solvolysis of ethyl fluoroformate (EtOCOF)*a* and ethyl chloroformate (EtOCOCl)*b* at various temperatures, and enthalpies (**Δ**H^≠^, kcal mol^−1^) and entropies (ΔS^≠^, cal mol^−1^ K^−1^) of activation.

Solvent*c* (%)	Temp.(°C)	10^4^ k_F_ (sec^−1^)	EtOCOF	ΔS^≠^_297.4°C_*d*	10^4^ k_Cl_ (sec^−1^)	EtOCOCl	ΔS^≠^_297.4°C_*d*
			ΔH^≠^_297.4°C_*_d_*			ΔH^≠^_297.4°C_*_d_*	
100MeOH	24.2	0.769±0.040	12.0±0.4	−36.9±1.4	0.824±0.009*e*	14.8±0.2	−27.5±0.8
	35.0	1.74±0.16			2.16±0.01		
	45.0				4.59±0.17		
	55.0	5.78±0.08			9.59±0.23		

100EtOH	24.2	0.128±0.010	12.6±0.1	−38.5±0.5	0.226±0.005*e*	14.6±0.3	−30.7±1.1
	35.0				0.528±0.028		
	45.0	0.544±0.020			1.15±0.01		
	55.0	1.05±0.03			2.54±0.08		

80EtOH	24.2	6.39±0.4	9.4±0.6	−41.5±2.0	0.731±0.006*e*	13.6±0.2	−31.8±0.5
	35.0	10.8±0.3			1.75±0.03		
	40.0	14.3±0.2					
	45.0	19.6±0.3			3.72±0.05		
	55.0				7.21±0.24		

70TFE	24.2	1.18±0.04	13.3±0.5	−31.7±1.5	0.0611±0.002*e*	18.5±0.2	−20.4±0.7
	45.0	4.47±0.19			0.503±0.005		
	50.0	7.78±0.13					
	55.0				1.30± 0.02		
	60.0				1.90±0.05		

70HFIP	24.2	2.38±0.19	14.0±0.4	−27.9±1.5			
	35.0	5.52±0.07					
	40.0	8.40±0.07					

^a^ Substrate concentration of 5.86×10^−3^ mol dm^−3^.

^b^ Substrate concentration of 5.12×10^−3^ mol dm^−3^.

^c^ 80% EtOH prepared on a volume/volume basis, at 25.0 °C and 70% TFE and 70% HFIP prepared on a weight/weight basis.

^d^ With associated standard error.

^e^ Values from ref. [19].

**Table 3.** The specific rate ratios (k_F_/k_Cl_) of solvolyses of alkyl haloformates in pure and binary solvents at various temperatures.

Solvent(%)*a*	ethyl*c*	*n*-propyl*d*	*i*-propyl*e*	*n*-octyl*f*	benzyl*g*	1-adamantyl*h*	2-adamantyl*i*
100EtOH	0.57	0.57	0.18	0.62	1.19	1.31×10^−17^	0.37
80EtOH	8.74	5.62	2.11	8.09	11.5	1.25×10 ^−3^	3.48
60EtOH	14.0		1.79	15.1	14.6*l*		3.01
100MeOH	0.93	0.75	0.39	0.95	1.78	5.91×10^−11^	0.42
90MeOH	4.82	-	1.76	-	7.18	-	2.40
80Me_2_CO	3.90	4.24*j*	0.53	2.86	5.89	-	0.65
70TFE*b*	19.3	7.72	0.067	10.2*k*	6.36	-	0.011

^a^ Unless otherwise indicated, on a volume/volume basis, at 25.0 °C, with the other component water.

^b^ Solvent prepared on weight/weight basis.

^C^ At 24.2 °C (this study).

^d^ At 40.0 °C [31].

^e^ At 40.0 °C [40].

^f^ At 24.2 °C [20].

^g^ At 25.0 °C [32].

^h^ At 50.0 °C [30].

^i^ At 25.0 °C [33].

^j^ For 70% acetone.

^k^ For 80% TFE.

^l^ For 70% ethanol.

**Table 4.** Correlation of the specific rates of solvolysis of ethyl fluoroformate and a comparison with the corresponding values for the solvolyses of other halogenoformate esters using the extended Grunwald-Winstein equation.

Substrate	Mech.*a*	n*b*	*lc*	*mc*	*cc*	R*d*	*l/m*
EtOCOF	A-E	21	1.51±0.20	0.85±0.11	−0.14±0.14	0.883	1.78
EtOCOF	A-E	17*e*	1.34±0.14	0.77±0.07	−0.06±0.10	0.942	1.74
EtOCOCl*f*	A-E	28	1.56±0.09	0.55±0.03	0.19±0.24	0.967	2.84
EtOCOCl*f*	I	7	0.69±0.13	0.82±0.16	−2.40±0.27	0.946	0.84
*n*-PrOCOF*g*	A-E	19	1.80±0.17	0.96±0.10	−0.01±0.11	0.940	1.88
*n*-PrOCOCl*h*	A-E	22	1.57±0.12	0.56±0.06	0.15±0.08	0.947	2.79
*n*-PrOCOCl*h*	I	6	0.40±0.12	0.64±0.13	−2.45±0.47	0.942	0.63
*i-*PrOCOF*i*	A-E	20	1.59±0.16	0.80±0.06	−0.12±0.05	0.957	1.99
*i-*PrOCOCl*j*	I	20	0.28±0.05	0.52±0.03	−0.12±0.05	0.979	0.54
*n*-OctOCOF*k*	A-E	23	1.80±0.13	0.79±0.06	0.13±0.34	0.959	2.28
BzOCOF*l*	A-E	16	1.57±0.20	0.76±0.08	−0.13±0.27	0.974	2.04
1-AdOCOF*m*	A-E	10	2.78±0.21	1.01±0.06	0.09±0.16	0.987	2.78
1-AdOCOF*m*	I	16	~0	0.70±0.01	−0.02±0.05	0.999	~0
2-AdOCOF*n*	A-E	17	1.92±0.15	0.84±0.06	−0.02±0.06	0.968	2.28

^a^ Addition-elimination (A-E) and ionization (I).

^b^ Number of solvent systems included in the correlation.

^c^ Using equation (1), with standard errors for *l* and *m* values and with the standard errors of the estimate accompanying the c values.

^d^ Correlation coeffient.

^e^ Omitting the four TFE-ethanol solvents.

^f^ Values from ref. [19].

^g^ Values from ref. [31].

^h^ Values from ref. [39].

^i^ Values from ref. [40].

^j^ Values from ref. [14].

^k^ Values from ref. [20].

^l^ Values from ref. [32].

^m^ Values from ref. [30].

^n^ Values from ref. [33].
